# Moderation of parental socioeconomic status on the relationship between birth health and developmental coordination disorder at early years

**DOI:** 10.3389/fped.2023.1020428

**Published:** 2023-03-15

**Authors:** Huynh-Truc Tran, Yu-Ting Tseng, Shuya Chen, Sheng K. Wu, Yao-Chuen Li

**Affiliations:** ^1^ Department of Physical Therapy & Graduate Institute of Rehabilitation Science, China Medical University, Taichung, Taiwan; ^2^ Department of Kinesiology, National Tsing Hua University, Hsinchu, Taiwan; ^3^ Research Center for Education and Mind Sciences, National Tsing Hua University, Hsinchu, Taiwan; ^4^ Department of Sport Performance, National Taiwan University of Sport, Taichung, Taiwan

**Keywords:** developmental coordination disorder, family factors, child health, early childhood, moderation

## Abstract

**Objective:**

This study investigated whether parental SES moderates the effect of birth health on Developmental Coordination Disorder (DCD) in preschool children.

**Methods:**

One hundred and twenty-two children aged 4 to 6 years were enrolled in the study. The Movement Assessment Battery for Children --2nd Edition (MABC-2) test was used to assess the motor coordination of children. They were preliminarily categorized into either the DCD (<=16th percentile, *n* = 23) or typically developing (TD) group (>16th percentile, *n* = 99) based on the testing results. All children in the DCD group were further confirmed to meet other diagnostic criteria of the DSM-V using the intellectual test and parental questionnaires. Moderation analysis was conducted using the PROCESS macro for SPSS, and 95% confidence intervals with a bootstrap procedure were calculated to identify the significant moderating effect.

**Results:**

Maternal education (unstandardized coefficient = 0.6805, SE = 0.3371, *p* < 0.05) and maternal employment status (unstandardized coefficient = 0.6100, SE = 0.3059, *p* < 0.05) were found to moderate the relationship between birth length and the probability of having DCD. Moreover, the relationship between birth weight and the probability of having DCD was moderated by the annual household income (unstandardized coefficient = −0.0043, SE = 0.0022, *p* < 0.05).

**Conclusion:**

The lower maternal education level and maternal unemployment strengthened the negative relationship between birth length and the probability of having DCD. Additionally, the negative relationship between birth weight and the probability of having DCD was statistically significant in high annual household salaries.

## 1. Introduction

Developmental coordination disorder (DCD) is one of the common neurodevelopmental disorders which has been recognized since the beginning of the 20th century due to poor motor coordination or clumsiness (1). DCD is estimated to impact around 6% of school-aged children (2) and notably interferes with children in accomplishing activities of daily living (e.g., buttoning, tying shoelaces, and using utensils), execution of motor skills at school (e.g., painting, handwriting, and sports), or performance in social envidronments (e.g., participating in playground activities and developing friendship with the peers) (2). Although motor coordination deficits are fundamental symptoms of DCD, this disorder could also lead to other non-motor problems, such as depression/anxiety (3–5), lower self-efficacy (6, 7), and lower life satisfaction (8).

A recent systematic review has shown that children born very preterm (before 26 weeks of gestation) and/or have very low birth weight (<1,500 grams) have the probability of having DCD 6 to 8 times higher than the term-born peers (9–12). In addition, lower Apgar scores have been found to be associated with minor motor, language, speech, and developmental impairments in school-aged children (13–15). As the advancement in neonatal care has led to the improvement in the survival rate of high-risk infants, such as low birth weight or low gestation age, these infants may experience challenges in motor function and be at greater risk for DCD later in their lives (16). Therefore, understanding the association between child birth health and the probability of developing DCD will be of importance from the perspective of preventive medicine.

In spite of the strong correlation between parental growth and child growth patterns (17), other contextual factors, such as parental socioeconomic status (SES), could also impact intrauterine and child development (18, 19). Prior research has showed the intertwined relationship between lower familial SES and poorer infant health, such as premature birth or low birth weight (20), and this adverse effect of low SES may begin prior to birth and exist from infancy to adulthood (18, 21). Due to poor prenatal care, poor nutrition during pregnancy, and other health problems, children from low SES families may be more likely to be born prematurely, having low birth weight and poor neurobehavioural development, such as poor cognitive abilities or poor social communication with peers (18, 22–24). More importantly, this detrimental long-term effect could predict poor physical and mental health during the childhood and adolescence. For example, a recent review has synthesized evidence from several cohort studies and summarized that children and/or adolescents from the low SES families are at greater risk for overweight/obesity, emotional and behavioural problems, and poor cardiovascular health (25). On the other hand, families with high SES could afford their children to access a variety of services, goods, and social interactions, and this could in turn lead to better child health (26).

The literature has indicated that families with low SES are more likely to have children who are uncoordinated, compared to their peers (27, 28). Specifically, the lower levels of parental education and family income have been reported to predict lower levels of motor development in children aged between 3 and 7 years (29). Compared to children from high SES families, those from low SES families were also more likely to be identified as motor developmental delay in a variety of cultural contexts, including Pakistan (30), the United States (31), and Brazil (32). Despite of scarce evidence, the existing findings have shown that lower parental SES could increase the risk of having children with DCD in the UK (33) and Israel (34). Nevertheless, there is a contradictory finding, suggesting that parents with higher education levels could have a higher chance of having children with DCD (35). Therefore, when parental SES may impact the occurrence of DCD, there are still inconsistent findings which need to be further investigated in more depth.

In order to early identify and intervene children with DCD, an enormous number of studies have investigated the potential perinatal or postnatal predictors for DCD to better understand the underlying mechanism (16). However, most of them have only focused on a single pathway or causation, rather a complicated mechanism that may simultaneously and comprehensively take into account multiple factors and their interactions (i.e., moderation) on DCD. Based on our literature review, aforementioned evidence may suggest that parental SES could serve as a moderator that buffers the impact of poor birth health on the probability of developing DCD.

By investigating this issue, our findings may help raise parental awareness toward DCD, formulate the diagnostic strategies for preschool children at risk of DCD, and identify the modifiable factors which may buffer the adverse effect of poor birth health on the occurrence of DCD. Therefore, the purpose of this study was to understand the differences in birth health and parental SES between children with and without DCD. Additionally, this study examined the moderation of parental SES on the relationship between birth health and DCD. It was hypothesized that parental SES would moderate the association between birth health and DCD.

## 2. Materials and methods

### 2.1. Participants

One hundred and twenty-two children aged 4 to 6 years were enrolled in the study. All children completed the assessment of motor coordination using the Movement Assessment Battery for Children—Second edition (MABC-2) test. Based on the testing results, 23 children (17 boys, 6 girls; 5.17 ± 0.54 years old) were categorized into the DCD group (<=16th percentile) and 99 children (47 boys, 52 girls; 5.16 ± 0.65 years old) in the typical development (TD) group (>16th percentile). Children in the DCD group were further assessed and confirmed based on the diagnostic criteria of the DSM-V. All measures used in this process were described below. Children in both groups had neither intellectual impairment (IQ < 70) nor neurological/movement disorders that may lead to motor deficits. Their caregivers (101 mothers and 19 fathers) were also invited to join the study. Written informed consents were obtained from children's parents.

### 2.2. Research design and procedure

This study was a secondary data analysis using the baseline data collected from the Stress, Motor coordination, and Activity Relationships in Taiwanese children (SMART) study, which was a prospective study approved by the Institute of Research Board of China Medical University (CRREC-108-021). In the SMART study, twenty-two preschools in Taichung City, Taiwan, were initially invited; eventually, six of them (27.3%) provided permission and agreed to participate in this study. The baseline data collection of the SMART study was conducted in 2020 (Wave 1) and 2021 (Wave 2). Each wave consisted of two phases. During Phase I, children's motor coordination and intelligence were evaluated using the MABC-2 test (36) and the Test of Nonverbal Intelligence - Fourth Edition (TONI-4) (37) at kindergartens, respectively. All families of children with DCD were invited to visit the lab to participate in the second phase of the SMART study. However, due to the impact of the COVID-19 pandemic, the recruitment of TD children was different between Wave 1 and Wave 2: families of TD children were conveniently selected in Wave 1, whereas those in Wave 2 were randomly selected. In the second phase, parents were requested to complete the parental questionnaire regarding parental SES and their children's birth health. All assessments were administered by trained research assistants. As the SMART study was a prospective study, all children and their parents were invited to re-visit the lab one year later for two consecutive years to receive the repeated assessment of all tests. As the SMART study is still ongoing, this study only used the baseline data for analysis.

### 2.3. Measures

#### 2.3.1. Developmental coordination disorder (DCD)

DCD was defined based on four diagnostic criteria of the Diagnostic and Statistical Manual of Mental Disorders, Fifth Edition (DSM-V) (2). The SMART study utilized the Movement Assessment Battery for Children-2 (MABC-2) test (36) to assess children's motor coordination. The MABC-2 was a standardized motor test to evaluate manual dexterity, aiming and catching, and balance in children aged between 3 and 16 years. It has been validated in Taiwanese preschool and school-aged children and showed excellent internal consistency and test-retest reliability (38). The age-appropriate testing items of the MABC-2 test were selected by the SMART study, and the one-on-one assessment was scheduled and conducted at kindergartens. As the DSM-V did not provide a clear cut-off point for motor impairments, motor difficulties were defined in this study if children scored at or below the 16th percentile on the MABC-2 test based on the international clinical practice recommendations (Criterion A of the DSM-V) (9). Children's parents were requested to complete the MABC-2 Checklist to confirm whether children's motor difficulties might impact their activities of daily living or academic tasks at preschool (Criterion B of the DSM-V).

As all children who participated in this study were aged between 4 and 6 years, they were in early developmental years based on the UNICEF definition, suggesting that early childhood spans the period up to 8 years of age (Criterion C of the DSM-V) (39). The Chinese version of the Test of Nonverbal Intelligence—Fourth Edition (TONI–4) with the Taiwanese norm was used to evaluate fluid intelligence in preschool children (37), and none of our participants was found to have intellectual impairment (i.e., IQ < 70). Additionally, parents were requested to complete the questionnaire to report their children's medical history and to confirm that children did not have any medical conditions which may lead to motor impairments (Criteria D of the DSM-V).

#### 2.3.2. Birth health

Birth health is the health condition of an infant at birth which is typically assessed by medical professionals immediately following delivery. As a variety of indicators could be used to measure birth health, this study retrospectively obtained data on birth weight, birth length, head circumference, gestation age, and the Apgar score at 5 min after birth from children's health booklets. The data on these variables were recorded by an obstetrician when each child was born. We further calculated the percentile of birth weight for gestation age (BWGA) based on the Taiwanese norms (40).

#### 2.3.3. Parental SES

Three main parameters were selected to determine the parental SES, including the annual household income, parental education level, and parental employment status (41). The parent who visited the lab with the child was requested to complete the questionnaire which included three specific questions asking parental SES, including the paternal and maternal highest level of education, paternal and maternal employment status, and the annual household income. There were three options for the educational level: Level 1 = high/ college/ technical school or lower; Level 2 = undergraduate; and Level 3 = graduate or higher. The employment status was categorized into six different situations: 1 = stay–at-home parent, 2 = working full-time for pay, 3 = working part-time for pay, 4 = working and parenting, 5 = looking for work, and 6 = retired. The annual household income was stratified into 12 levels: Level 1 = below NTD (New Taiwan Dollar) 300,000, Level 2 = NTD 300,000–499,999; Level 3 = NTD 500,000–699,999; Level 4 = NTD 700,000–899,999; Level 5 = NTD 900,000–1,099,999; Level 6 = NTD 1,100,000–1,299,999; Level 7 = NTD 1,300,000–1,799,999; Level 8 = NTD 1,800,000–2,299,999; Level 9 = NTD 2,300,000–2,799,999; Level 10 = NTD 2,800,000—3,299,999; Level 11 = NTD 3,300,000–3,999,999; Level 12 = above NTD 4,000,000.

#### 2.3.4. Statistical analysis

##### 2.3.4.1. Data management

In order to deal with the potential bias during data analysis, we have made some changes in the category of parental employment status and the annual household income, respectively. In the parental employment status, we combined the situation 1, 5, 6 into the category of “not having a job”, whereas the situation 2, 3, 4 were combined into “having a job”. In terms of the annual household income, we calculated the average income between the lowest and highest number in each level. *For example*, the level 1 = (0 + 300,000)/2 = 150,000. As the result, level 2 = 400,000; level 3 = 600,000; level 4 = 800,000; level 5 = 1,000,000; level 6 = 1,100,000; level 7 = 1,500,000; level 8 = 2,050,000; level 9 = 2,550,000; level 10 = 3,050,000; level 11 = 3,650,000; and level 12 = 4,000,000. Subsequently, the amount of the annual household income of each level was divided by 100,000 while conducting the moderation analyses.

It is also worth noting that, although there were specific cut-off points for some of birth health variables which were used to differentiate health status (e.g., low birth weight defined as <2,500 g, preterm birth as <37week of gestation age, or high-risk infant as <7 of Apgar score) (42–44), this study did not dichotomize these birth health variables into “typical” and “abnormal”. Instead, this study has used the continuous variables to represent these birth health outcomes as DCD may not only occur in those children with “definitively” poor birth health and the continuum of child development should be highlighted.

##### 2.3.4.2. Descriptive data analysis and correlations

Descriptive statistics were conducted using SPSS for Windows ver.26. All variables were presented in either mean ± standard deviation (SD) or N (%). To investigate the differences in sex, parental education level, and parental employment status between the DCD and TD groups, the Chi-square test was performed. In addition, the Mann-Whitney U test was utilized to examine the differences in the age, birth weight, birth length, head circumstance, gestation age, Apgar score at 5 min, and the annual household income between the DCD and TD groups. Furthermore, the correlation analysis between each variable of children's birth health and each variable of parental SES was performed using the Spearman's rank correlation coefficient. According to the Cohen's work (1988, 1992), the correlation coefficient (i.e., *r*) was one type of effect size that could be used to indicate small (*r* = .10), medium (*r* = .30), or large (*r* = .50) effect (45, 46). However, a recent study suggested that Cohen's guideline may overestimate effect sizes; thus, this study alternatively used *r* = .12,.20, and.32 to represent small, medium, and large effects, respectively (47).

##### 2.3.4.3. Moderation analysis

**Figure 1:**
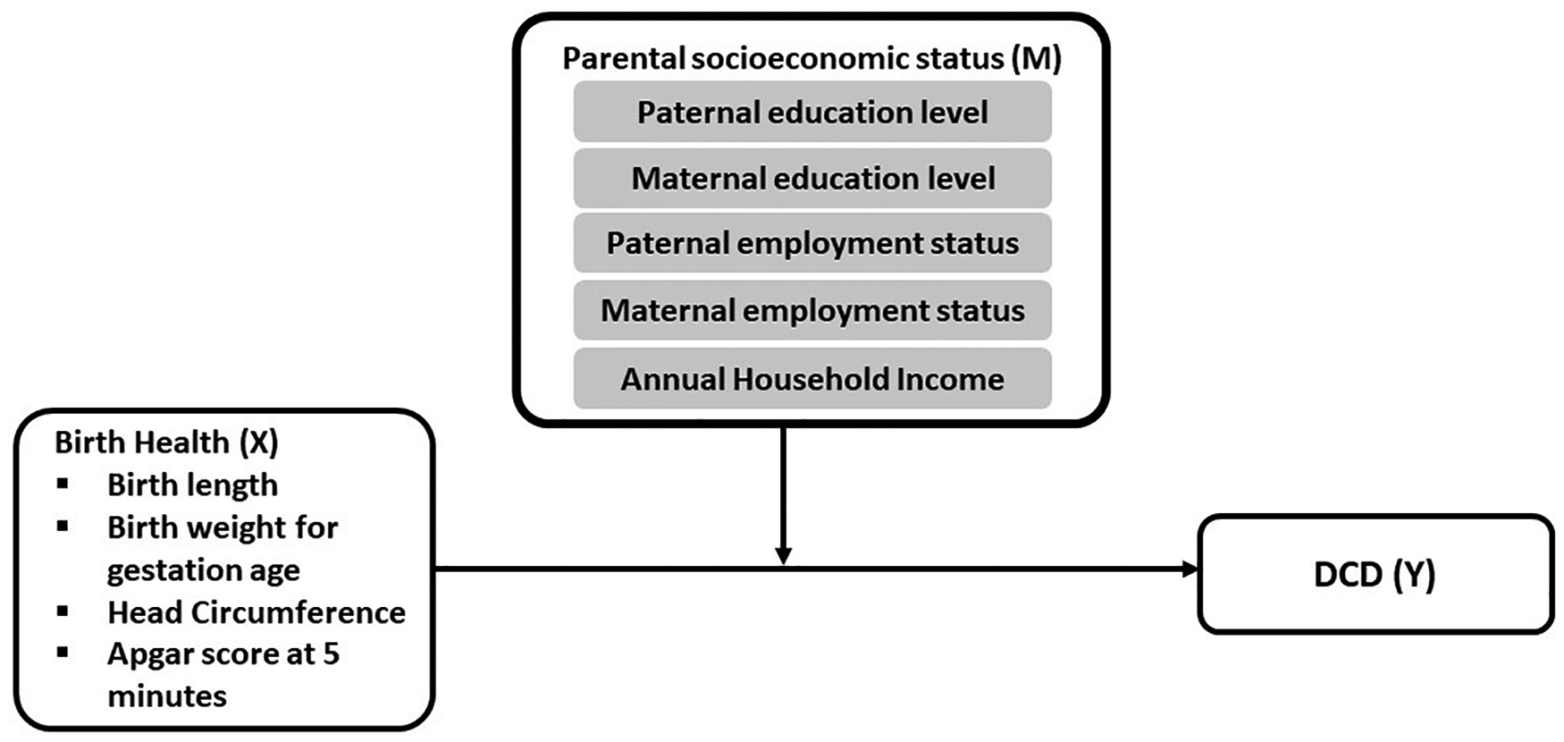
A conceptual diagram of a moderation model tested in this study.

The moderation analysis was conducted using the PROCESS software macro for SPSS (48). In the current study, we tested whether the effect of a child's birth health (independent variable, X) on DCD (dependent variable, Y) may be moderated by parental SES (moderator, W) (see Figure 1). The independent variables included birth length, BWGA, head circumference, and Apgar score at 5 min after birth, whereas the moderators included paternal and maternal educational levels, paternal and maternal employment status, and annual household income. In order to maximize the sample size for each tested model, this study separately examined the effect of each moderator. As a result, a total of twenty moderation models (4 birth health variables * 5 parental SES variables) were created and tested. The default Model 1 in PROCESS was chosen, and 95% confidence intervals (CI) were calculated with a 10,000 bootstrap procedure to identify the significant moderating effect. Furthermore, taking into account the potential impact of children's sex, age, and gestation age on the independent variables (i.e., birth health variables) and the dependent variable (i.e., DCD) (16, 49), these three variables were treated as covariates in all models. However, as BWGA has considered the potential influence of gestation age, only sex and age were covariates in the models with BWGA as the independent variable. The statistically significant moderating effect was defined if zero was not included by 95% CI. The result would be further graphed to interpret the moderating effect of a specific parental SES variable on the relationship between the birth health variables and the probability of having DCD.

## 3. Results

### 3.1. Overview of participants

Baseline demographic information was shown in Table 1. There was a significant sex difference between the two groups (*x*^2^ = 5.231, df = 1, *p* = 0.022), indicating significantly more boys in children with DCD (boys: girls = 2.83:1 in the DCD group and nearly 1:1 in the TD group). There were also significant group differences in the total test score of the MABC-2, demonstrating that children had significantly poorer motor coordination (*p* < 0.001).

**Table 1:** Baseline characteristics of the study population [mean ± SD or *n* (%)].

	DCD group	TD group	*p* value
	(*N* = 23)	(*N* = 99)	
Sex (girls)	6 (26.1%)	52 (52.5%)	0.022
Age (years old)	5.17 ± 0.54	5.16 ± 0.65	0.963
TONI-4	98.00 ± 10.82	99.24 ± 9.21	0.839
MABC-2	55.78 ± 10.34	85.89 ± 9.34	<0.001
Birth length (cm)	49.25 ± 2.60	49.98 ± 2.40	0.192
Birth weight (gram)	2891.00 ± 457.04	3030.04 ± 406.37	0.145
Head circumference (cm)	32.57 ± 1.33	33.22 ± 5.39	0.087
Gestation age (weeks)	38.38 ± 2.18	38.24 ± 3.27	0.734
Apgar score at 5 min	9.11 ± 0.83	9.32 ± 0.56	0.395
Body weight for gestation age (percentile)	43.00 ± 33.38	52.18 ± 28.92	0.238
Paternal age (years old)	39.47 ± 5.14	39.01 ± 9.60	0.853
Maternal age (years old)	36.43 ± 9.06	37.75 ± 4.51	0.430
**Paternal educational level**
High school/ College/ Technical school	10 (47.6%)	27 (30.7%)	0.320
Undergraduate	8 (38.1%)	41 (46.6%)	
Graduate	3 (14.3%)	20 (22.7%)	
**Maternal education level**
High school/ College/ Technical school	9 (40.9%)	27 (28.4%)	0.390
Undergraduate	9 (40.9%)	54 (56.8%)	
Graduate	4 (18.2%)	14 (14.7%)	
**Paternal employment status**
Not having job	2 (10.0%)	6 (6.7%)	0.637
Having job	18 (90.0%)	83 (93.3%)	
**Maternal employment status**
Not having job	6 (30.0%)	35 (37.6%)	0.519
Having job	14 (70.0%)	58 (62.4%)	
Annual household income (NDT)	1,211,000 ± 1,083,000	932,000 ± 506,000	0.981

MABC-2, The total score of Movement Assessment Battery for Children—Second edition; TONI-4, The total score of the Test of Nonverbal Intelligence—Fourth Edition.

Compared to the TD peers, children with DCD had lower birth weight, shorter birth length, smaller head circumference, lower gestation age, and Apgar score at 5 min, and lower percentile of BWGA; however, there were no significant group differences in these variables (all *p*'s > 0.05). In addition, there were no significant differences in parental SES variables between groups (all *p*'s > 0.05).

### 3.2. The correlation between birth health and parental SES

As shown in Table 2, among the birth health variables, children's birth length was significantly, positively associated with the other variables with medium to large effects (*r* = 0.261–0.696, all *p*'s < 0.01), except Apgar score (*r* = 0.122, *p* > 0.05). Birth weight was also significantly, positively associated with BWGA with a large effect (*r* = 0.732, *p *< 0.01) and head circumference with a small effect (*r* = 0.196, *p *< 0.05). There were also significant associations between gestation age and Apgar score at 5 min with a medium effect (*r* = 0.217, *p *< 0.05) and between BWGA and head circumference with a large effect (*r* = 0.310, *p *< 0.01).

**Table 2:** The correlation between birth health and parental SES variables.

	Birth health						Parental SES				
	1	2	3	4	5	6	7	8	9	10	11
1. Birth length	1										
2. Birth weight	0.696**	1									
3. Gestation age	0.261**	0.113	1								
4. Birth weight for gestation age	0.500**	0.732**	−0.133	1							
5. Head circumference	0.279**	0.196*	−0.009	0.310**	1						
6. Apgar score at 5 min	0.122	0 l.104	0.217*	−0.138	−0.104	1					
7. Paternal education level	−0.094	0.019	0.006	−0.168	−0.085	0.125	1				
8. Maternal education level	−0.013	0.113	0.171	−0.109	0.117	0.085	0.577**	1			
9. Paternal employment status	0.075	0.127	0.119	0.002	0.122	0.231*	0.147	0.210*	1		
10. Maternal employment status	−0.165	−0.156	−0.038	−0.146	0.000	0.199	0.095	0.227*	−0.045	1	
11. Annual household income	−0.055	0.115	0.159	−0.162	0.198	0.125	0.497**	0.546**	0.161	0.230*	1

* *p* < 0.05.

** *p* < 0.01; SES, socioeconomic status.

In terms of the parental SES variables, the paternal educational level was significantly, positively associated with maternal educational level with a large effect (*r* = 0.577, *p *< 0.01) and the annual household income with a large effect (*r* = 0.497, *p *< 0.01), whereas the maternal educational level was significantly associated with paternal with a medium effect (*r* = 0.210, *p *< 0.05) and maternal employment status with a medium effect (*r* = 0.227, *p *< 0.05), as well as the annual household income with a large effect (*r* = 0.546, *p *< 0.01). Furthermore, maternal employment status was significantly, positively associated with the annual household income with a medium effect (*r* = 0.230, *p *< 0.05). When the associations between birth health and parental SES variables were investigated, only the Apgar score at 5 min demonstrated a significantly positive association with the paternal employment status with a medium effect (*r* = 0.231, *p *< 0.05).

### 3.3. Moderation of parental SES

#### 3.3.1. On the relationship between birth length and DCD

**Figure 2:**
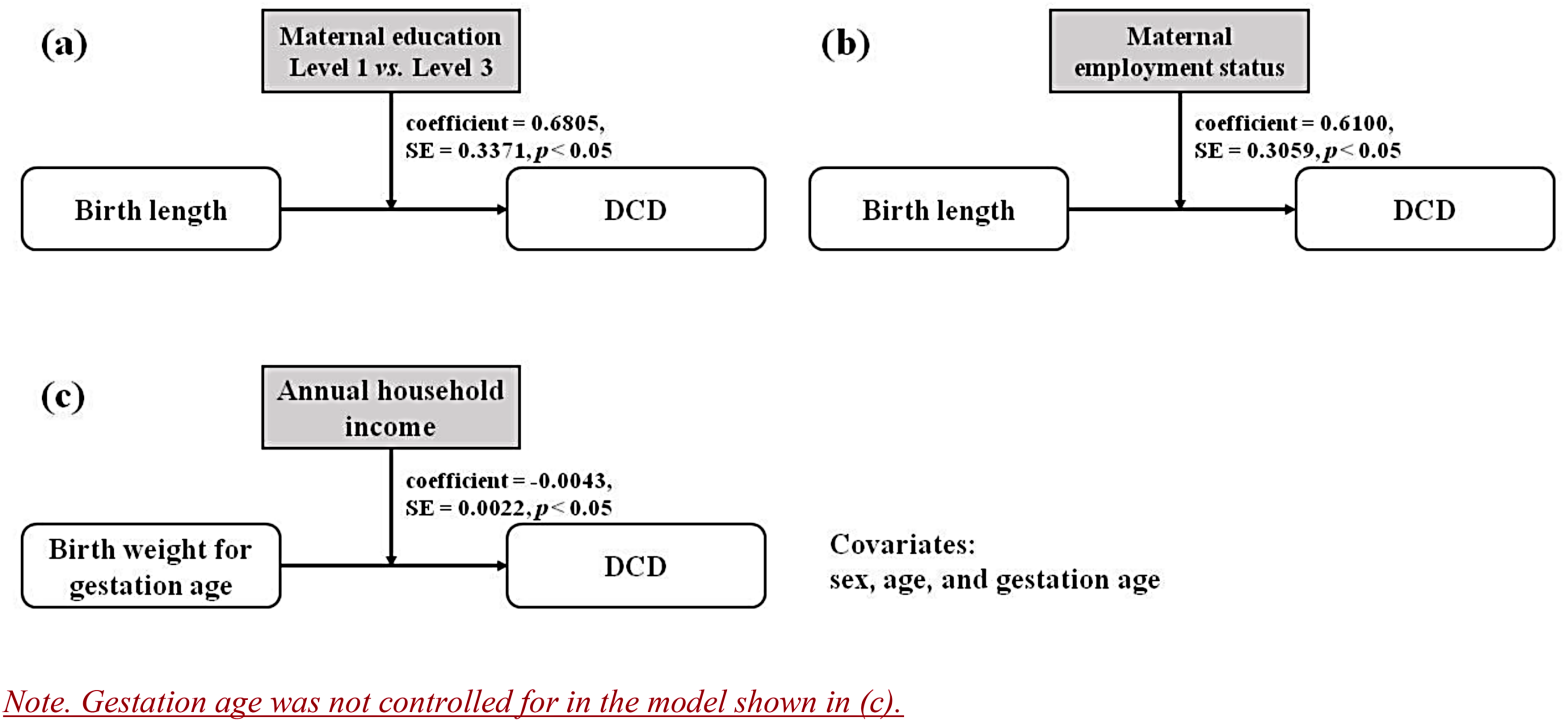
Models with the significant moderating effect of parental SES.

**Figure 3:**
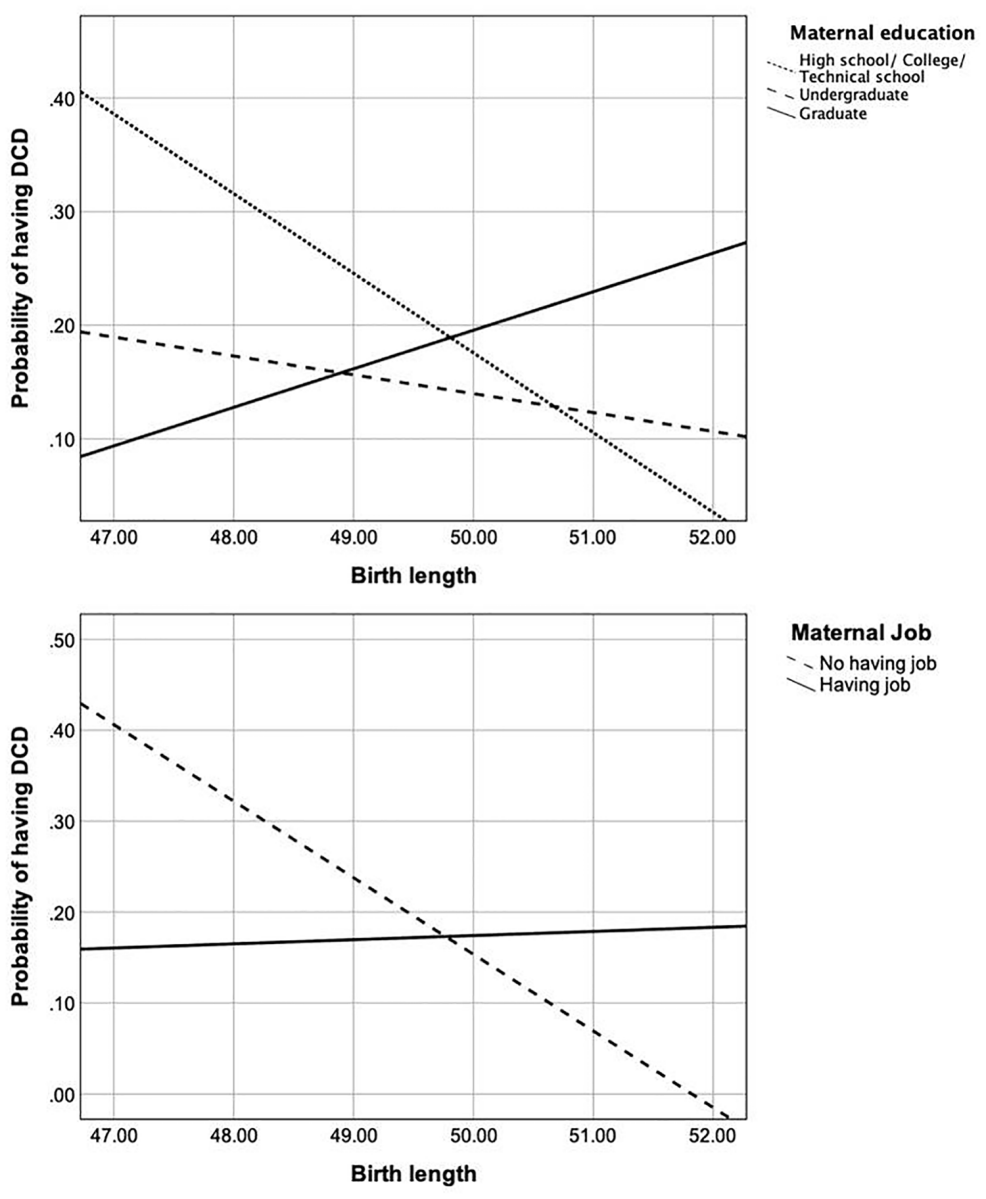
The relationship between birth length and the probability of having DCD in different situations of maternal educational level (upper graph) and employment status (lower graph).

There were two significant moderating effects on the relationship between birth length and DCD. This relationship was moderated by maternal educational level, specifically between Level 1 (i.e., high/college/technical school or lower) and Level 3 (i.e., graduate or higher) [unstandardized coefficient = 0.6805, SE = 0.3371, *p* < 0.05; Figure 2(a) and Supplementary Table S1]. In order to better understand the relationships in different conditions (i.e., educational levels), the result was graphed. As we could see in Figure 3, in children whose mother educational level was Level 1 (i.e., high/ college/ technical school or lower) and Level 2 (i.e., undergraduate), there was a negative correlation between birth length and the probability of having DCD, whereas this association pattern was reverse (i.e., positive correlation) in those whose mother educational level was Level 3 (i.e., graduate or higher).

Similarly, a significant moderation effect was found for the maternal employment status on the relationship between birth length and DCD [unstandardized coefficient = 0.6100, SE = 0.3059, *p* < 0.05; Figure 2(b) and Supplementary Table S1]. The detailed result indicated that if the mother was unemployed, the lower birth length was associated with the greater probability of having DCD, while in the employed mothers, there seemed to be no association between birth length and the probability of having DCD (Figure 3).

#### 3.3.2. On the relationship between birth weight for gestation age and DCD

**Figure 4:**
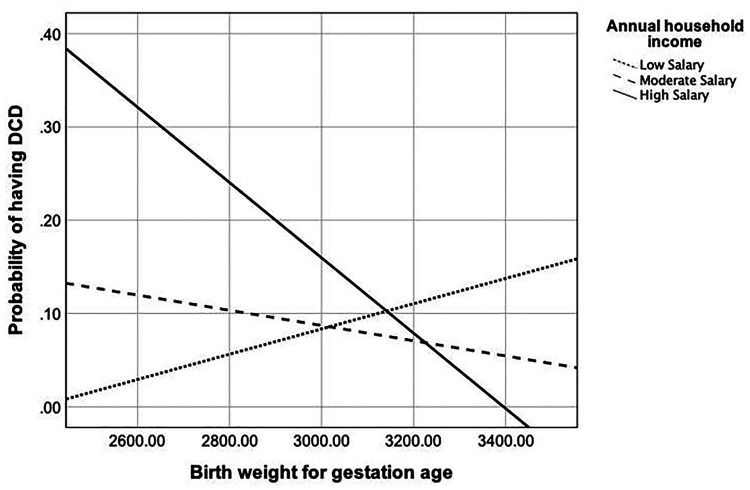
The relationship between birth weight and the probability of having DCD in different levels of the annual household income.

As shown in Supplementary Table S2, only the annual household income was found to moderate the relationship between birth weight and DCD [unstandardized coefficient = −0.0043, SE = 0.0022, *p* < 0.05, Figure 2(c)]. Figure 4 showed that in the condition of lower annual household income, birth weight was positively associated with the probability of having DCD, while in the conditions of medium and higher annual household income, a negative association was found. Nevertheless, the moderating effects of the remaining parental SES factors were not found to be significant on the association between birth weight and DCD.

#### 3.3.3. On the relationship between head circumstance and DCD

Parental SES variables were not found to significantly moderate the relationship between head circumference and DCD (see Supplementary Table S3).

#### 3.3.4. On the relationship between birth length and DCD

In a similar manner, there were no moderating effects of parental SES variables on the Apgar score at 5 min and DCD (see Supplementary Table S4).

## 4. Discussion

To the best of our knowledge, this is the first study to examine the potential moderating effect of parental socioeconomic status on the relationship between birth health and developmental coordination disorder during early childhood. Our findings lend partial support to our hypotheses, indicating that parental SES may moderate the relationship between birth health and DCD in some specific circumstances. Specifically, the relationship between birth length and the probability of having DCD could be separately moderated by the maternal educational level and employment status. Additionally, the annual household income may moderate the relationship between birth weight and the probability of having DCD. While a limited number of studies could be referenced to support our results, potential interpretation for each significant moderating effect was provided below.

### 4.1. The moderation of maternal education on the relationship between birth length and DCD

While more attention has been drawn to the effect of birth weigh on DCD (16), it is surprising that the importance of birth length is less investigated. However, prior research has shown that birth length or small-for-gestational-age could be also associated with child health and development (50); specifically, the shorter birth length is often considered as a predictor of child development, such as poorer gross motor, language and social performance, during childhood. There was a significant moderation of maternal educational level (Level 1 *vs*. Level 3) on the relationship between birth length and DCD, and this moderating effect showed different patterns for these two levels. Specifically, in a family in which the mother had lower educational level (i.e., high school/ college/ technical school or undergraduate), the shorter birth length was associated with the higher probability of having DCD. A lack of research has investigated how maternal educational level may impact children's birth length. However, a recent cohort study recruiting Canadian mother-father-offspring triads has identified a stronger association of small-for-gestation-age with maternal education than that with paternal education (51). Specifically, mothers with the lower educational level (i.e., high school) have been found to be at significantly greater risk for having babies with small-for-gestation-age, compared to those with the higher educational level (i.e., college or university), whereas paternal education is associated with none of infant health outcomes (51). Moreover, previous studies have shown that women having less than eight years of formal education are 1.5 times more likely to have infants with low birth weight (52). Due to a significantly positive correlation between birth weight and birth length in this study, this may also indirectly support our finding (53). Therefore, as shorter birth length is associated with the higher probability of having DCD if mothers have the lower educational level, more attention should be drawn to birth health for expectant mothers with the lower educational level to prevent their children from developing DCD.

It is worth noting that while the maternal education level is higher (i.e., graduate), the relationship between birth length and the probability of having DCD would be reversed, showing that longer birth length is associated with a higher probability of having DCD. Evidence has demonstrated that an increase in parents' schooling, specifically maternal schooling, would result in better infant health (54). However, a mother with a higher educational level has been found to have lower fertility intentions (55), and parents with higher educational levels may be prone to have a pregnancy at a later age (35). As a result, this might increase the possibility to have only one child, which has been identified as a significant predictor of DCD, due to a lack of daily interaction with a sibling in the family and the overprotection of parents of their only child (56). On the other hand, better-educated mothers may have a better awareness of the negative consequences associated with poor birth health and be more likely to adopt compensatory behaviours, such as devoting more resources to less-endowed children, to minimize the adverse outcomes (57). This could interpret why the probability of having DCD is lower when birth length is shorter and the maternal educational level is higher in this study.

### 4.2. The moderation of maternal employment status on the relationship between birth length and DCD

Although the paternal and maternal employment status may be inter-related (58), the association between birth length and the probability of having DCD was moderated by the maternal employment status, indicating that maternal unemployment may play a buffering role in further decreasing the risk for having DCD when their children have better birth health (i.e., longer birth length *herein*). Maternal employment status could affect many aspects of the development outcome of a child, including motor development (59). If a mother is unemployed, she may have more time spent with her children, which may benefit child development. Notably, while some mothers are willing to give up their job and stay at home to spend time with their children, others may be unwillingly unemployed. The latter may posit mothers to be at a higher level of stress associated with financial and employment uncertainty and the time spent in job search; consequently, this may deteriorate the quantity and quality of parent-child time, specifically for mothers (60). Therefore, unemployed mothers may be at increased risk for psychological problems which could in turn initiate a detrimental effect on the mother-child interaction and increase children's anxious and depressed behaviors (61), which may lead to poor gross motor performance that is one of the characteristics of DCD (62). Furthermore, although our study did not identify the significant effect of birth length on DCD in employed mothers, prior research has found that more than one-third of mothers who continue working during pregnancy report increased job stress which may result in a greater risk of having a new-born baby with poor birth health, such as low birth weight, and is considered as one of stronger predictors for DCD (63, 64).

Interestingly, although the aforementioned studies emphasized the crucial role of birth weight in child development and its association with maternal education or maternal employment status, our findings support that birth length might be a more important predictor of DCD. It is not clear whether this discrepancy could be attributed to more missing data in birth weight in this study (*see* Supplementary Tables S1, S2), or simply because birth length is not recorded during the delivery and is considered as being less important than birth weight (65, 66). Therefore, more rigorous studies enrolling a large sample are warranted to simultaneously evaluate and understand the roles of birth weight and length.

More importantly, our findings highlight the more significant contribution of mothers to infant health and child development than that of fathers, which is consistent with prior research (58). Despite the fact that there is support for the importance of paternal engagement in infant health (19, 67), empirical evidence is relatively scarce. Some studies that investigated the effect of paternal and maternal education on birth health have reported that the effect of fathers appears approximately half that of mothers, while others have asserted the equal effects (68, 69) or a stronger effect of fathers (70). As this study was conducted in the Asian cultural context where parental roles may be different from those in other countries, further research may be needed to investigate the cultural difference of parental roles in child health.

### 4.3. The moderation of annual household income on the relationship between birth weight for gestation age and DCD

The patterns showed that the higher annual household income may have a protective effect on children's risk for DCD related to BWGA. In families with the higher annual household income, the probability of having DCD is higher than it in families with the lower annual household income if a child was born with the lower BWGA. In spite of a lack of direct evidence, we speculate that this may be related to the mother's age at pregnancy as prior research has found that the employed mothers who have the higher income may be pregnant at an older age which could increase the risk of childhood neurodevelopmental disorders (71). Furthermore, if a child was born with the higher BWGA, the economic disadvantage might increase the probability of having DCD since the parents may not be able to afford sufficient stimuli for child development of their children (72).

Our result is consistent with previous research suggesting that many children from low SES families lack access to the same resources and experiences, compared to children from high SES families, thus putting them at greater risk for developmental problems (73). On the other hand, the higher household income, representing the proxy of higher-quality parenting behaviours and a stable social environment (74), could be beneficial for the development and maturation of the cerebellum that has been found to be associated with children's performance on motor coordination (75). However, this finding seemingly stands in contrast to the “strategic investment” argument (76) that disadvantaged families concentrate resources on higher-ability children in an effort to reduce risk and maximize expected returns to human-capital investments, while advantaged families, on the other hand, adopt compensatory strategies, devote more resources to less-endowed children. This may be because, compared to advantaged families, “problematic” children from disadvantaged families may impose more financial and psychological burdens on their parents; as a results, these disadvantageous parents tend to spend more time with “easier” children (57).

Another explanation may be related to the effect of parental overprotection which might be stronger for families with the medium and higher SES (77) and be more often seen in boys (78) which may have a higher incidence of DCD (7, 79, 80). Yet, the higher annual household income was associated with the greater possibility of higher maternal educational level (*see* Table 2), which is the condition where the better child health (i.e., longer birth length) have been previously found to be associated with the higher probability of having DCD. This has led to controversial findings in this study. One of the possibilities may be the combination of paternal income and maternal income into the same variable of the household income which limits our ability to examine their influences, respectively. As a study in Canada has demonstrated that the contribution of women is approximately 22% to 33% of the annual household income (81), it is warranted to investigate the underlying impact of paternal and maternal contribution, respectively.

### 4.4. Limitations and future directions

While this study has identified specific moderators and the potential family who may be at greater risk for having DCD, there are a few limitations that need to be addressed. First, as early life and current SES may differently contribute to birth health and child development, the cross-sectional study design of the current study limits our ability to interpret the causal relationships between the studied variables. Further longitudinal studies will be needed. Second, there are lots of missing data on parental SES and children's birth health variables (at a maximum of 25%). This may lead to insufficient statistical power and generalizability of our results. Our results could be also biased by the unbalanced sample sizes between the two groups. This was due to the outbreak of the COVID-19 pandemic which hindered us from entering the preschools and conducting data collection. Therefore, we were unable to recruit more young children with DCD. However, as the purpose of this study was to conduct the moderation analysis, our sample size was considered as sufficient and appropriate. Third, the parental employment status used in the study does not measure the aspect of employment stability, such as the consistency in employment over time, the number of job transitions, or length of time in the same job, as the maternal employment instability may be linked with poorer child health outcomes (82). In addition, other factors or reasons related to unemployment, e.g., wage, quality, or benefits of jobs, all of which may affect child development (83), were not measured in the current study. Future research may use more detailed data collection methods to better understand the moderating effect of parental employment status. The final concern is the classification of the educational level. Our study categorized parental educational level into three levels, which may be different from the category in other studies. As there is no consensus regarding the classification of the educational level, this leads to the difficulty to compare our results to other studies. There are other factors, such as maternal age (84), marital status (85), parental psychology (86), and parental behaviour (87), which may affect birth health and development, but are not included in the current study. Therefore, we recommend future studies replicate our concept while controlling for the above factors to provide more valuable information into the nature of the associations that we have found.

## 5. Conclusions

In summary, this study shed a light on the underlying mechanism of DCD. Our results confirm that the shorter birth length is specifically associated with the increased probability of having DCD in preschool children whose mothers have lower educational levels. Similarly, an unemployed mother may strengthen the negative relationship between birth length and the probability of having DCD. Nevertheless, the positive association between birth weight and the probability of having DCD was reversed in families with higher annual household incomes. Moreover, this study also provides an important practical implication for paediatricians who would be able to early identify the child who is at greater risk of having DCD based on their birth health and parental SES, especially mothers. Furthermore, as our finding may help parents be more aware of early characteristics of DCD, they could raise the concerns to their child's pediatrician earlier if the child demonstrates any difficulty in motor coordination. Additionally, our results suggest insight into whether policies should in advance target those high-risk families for having DCD and provide the corresponding intervention based on familial SES.

## Data Availability

The original contributions presented in the study are included in the article/**Supplementary Material**, further inquiries can be directed to the corresponding author.
