# Supplementary material for: Moderation of parental socioeconomic status on the relationship between birth health and developmental coordination disorder at early years
Source: Front Pediatr. 2023 Mar 15;11:1020428. doi: 10.3389/fped.2023.1020428 (PMC10050449; doi:10.3389/fped.2023.1020428)
Supplement: Supplementary file 1 [file Datasheet1.docx]

Supplementary Material

# Supplementary Figures and Tables

## Supplementary Tables

**Table S1.** The moderating effect of parental SES on the relationship between birth length and DCD, controlling for children’s age, sex, and gestation age

| **Moderator** | | **Sample size** | **Coefficients (SE)** | **95% Bootstrap Confidence Interval** | |
| --- | --- | --- | --- | --- | --- |
|  |  |  |  | **Lower limit** | **Upper limit** |
| Paternal Education | Level 1 vs. Level 2 | 100 | 0.0793 (0. 2390) | -0.3891 | 0.5477 |
|  | Level 1 vs. Level 3 | 100 | 0.5628 (0.4129) | -0.2464 | 1.3719 |
|  | Level 2 vs. Level 3 | 100 | 0.4834 (0.4122) | -0.3244 | 1.2913 |
| Maternal Education | Level 1 vs. Level 2 | 105 | 0.2868 (0.2655) | -0.2336 | 0.8071 |
|  | Level 1 vs. Level 3 | 105 | **0.6805 (0.3371)*** | 0.0198 | 1.3412 |
|  | Level 2 vs. Level 3 | 105 | 0.3937 (0.3078) | -0.2096 | 0.9971 |
| Paternal Employment Status | | 100 | 25.3723 (843.6978) | -1628.2450 | 1678.9897 |
| Maternal Employment Status | | 102 | **0.6100 (0.3059)*** | 0.0105 | 1.2096 |
| Annual Household Income | | 97 | 0.0318 (0.0225) | -0.0123 | 0.0758 |

* p <0.05; SES: socioeconomic status.

Note. Level 1: high/ college/ technical school or lower; Level 2: undergraduate; Level 3: graduate or higher.

**Table S2.** The moderating effect of parental SES on the relationship between by birth weight for gestation age and DCD, controlling for children’s age, and sex

| **Moderator** | | **Sample size** | **Coefficients (SE)** | **95% Bootstrap Confidence Interval** | |
| --- | --- | --- | --- | --- | --- |
|  |  |  |  | **Lower limit** | **Upper limit** |
| Paternal Education | Level 1 vs. Level 2 | 93 | -0.0228 (0.0218) | -0.0654 | 0.0199 |
|  | Level 1 vs. Level 3 | 93 | 0.0057 (0.0303) | -0.0537 | 0.0651 |
|  | Level 2 vs. Level 3 | 93 | 0.0285 (0.0317) | -0.0336 | 0.0906 |
| Maternal Education | Level 1 vs. Level 2 | 97 | -0.0071 (0.0208) | -0.0479 | 0.0336 |
|  | Level 1 vs. Level 3 | 97 | -0.0130 (0.0317) | -0.0750 | 0.0491 |
|  | Level 2 vs. Level 3 | 97 | -0.0058 (0.0312) | -0.0670 | 0.0553 |
| Paternal Employment Status | | 93 | 0.0068 (0.0263) | -0.0447 | 0.0583 |
| Maternal Employment Status | | 94 | -0.0266 (0.0270) | -0.0794 | 0.0263 |
| Annual Household Income | | 90 | **-0.0043 (0.0022)*** | -0.0086 | -0.0001 |

**p* <0.05; SES: socioeconomic status.

Note. Level 1: high/ college/ technical school or lower; Level 2: undergraduate; Level 3: graduate or higher.

**Table S3.** The moderating effect of parental SES on the relationship between head circumference and DCD, controlling for children’s age, sex, and gestation age

| **Moderator** | | **Sample size** | **Coefficients (SE)** | **95% Bootstrap Confidence Interval** | |
| --- | --- | --- | --- | --- | --- |
|  |  |  |  | **Lower limit** | **Upper limit** |
| Paternal Education | Level 1 vs. Level 2 | 92 | 0.0140 (0.4140) | -0.7975 | 0.8254 |
|  | Level 1 vs. Level 3 | 92 | 0.4104 (0.3876) | -0.3493 | 1.1701 |
|  | Level 2 vs. Level 3 | 92 | 0.3964 (0.3248) | -0.2401 | 1.0329 |
| Maternal Education | Level 1 vs. Level 2 | 97 | 0.2180 (0.3135) | -0.3964 | 0.8323 |
|  | Level 1 vs. Level 3 | 97 | -0.2038 (0.6687) | -1.5144 | 1.1069 |
|  | Level 2 vs. Level 3 | 97 | -0.4217 (0.5940) | -1.5859 | 0.7424 |
| Paternal Employment Status | | 92 | 0.5036 (0.9900) | -1.4368 | 2.4440 |
| Maternal Employment Status | | 94 | 0.4228 (0.4017) | -0.3644 | 1.2100 |
| Annual Household Income | | 91 | 0.0139 (0.0233) | -0.0317 | 0.0595 |

SES: socioeconomic status.

Note. Level 1: high/ college/ technical school or lower; Level 2: undergraduate; Level 3: graduate or higher.

**Table S4.** The moderating effect of parental SES on the relationship between Apgar score at 5 minutes and DCD, controlling for children’s age, sex, and gestation age

| **Moderator** | | **Sample size** | **Coefficients (SE)** | **95% Bootstrap Confidence Interval** | |
| --- | --- | --- | --- | --- | --- |
|  |  |  |  | **Lower limit** | **Upper limit** |
| Paternal Education | Level 1 vs. Level 2 | 77 | 0.2713 (0.9891) | -1.6672 | 2.2098 |
|  | Level 1 vs. Level 3 | 77 | 0.3922 (1.8099) | -3.1551 | 3.9396 |
|  | Level 2 vs. Level 3 | 77 | 0.1209 (1.7314) | -3.2725 | 3.5143 |
| Maternal Education | Level 1 vs. Level 2 | 81 | 1.0640 (1.0187) | -0.9326 | 3.0606 |
|  | Level 1 vs. Level 3 | 81 | 2.0894 (1.6888) | -1.2205 | 5.3994 |
|  | Level 2 vs. Level 3 | 81 | 13.5388 (736.1619) | -1429.3120 | 1456.3895 |
| Paternal Employment Status | | 77 | 1.0254 (1.6721) | -2.2518 | 4.3026 |
| Maternal Employment Status | | 80 | -0.9824 (1.0051) | -2.9524 | 0.9876 |
| Annual Household Income | | 75 | 0.1847 (0.1135) | -0.0377 | 0.4072 |

SES: socioeconomic status.

Note. Level 1: high/ college/ technical school or lower; Level 2: undergraduate; Level 3: graduate or higher.
